# Gene Banks as Reservoirs to Detect Recent Selection: The Example of the Asturiana de los Valles Bovine Breed

**DOI:** 10.3389/fgene.2021.575405

**Published:** 2021-02-02

**Authors:** Simon Boitard, Cyriel Paris, Natalia Sevane, Bertrand Servin, Kenza Bazi-Kabbaj, Susana Dunner

**Affiliations:** ^1^GenPhySE, Université de Toulouse, INRA, INPT, INP-ENVT, Castanet-Tolosan, France; ^2^Dpto. Animal Production, Facultad de Veterinaria, Universidad Complutense de Madrid, Madrid, Spain; ^3^GABI, INRAE, AgroParisTech, Université Paris-Saclay, Jouy-en-Josas, France; ^4^SIGENAE, INRA, Jouy-en-Josas, France

**Keywords:** cattle, gene banks, time series, nSL, selection signatures

## Abstract

Gene banks, framed within the efforts for conserving animal genetic resources to ensure the adaptability of livestock production systems to population growth, income, and climate change challenges, have emerged as invaluable resources for biodiversity and scientific research. Allele frequency trajectories over the few last generations contain rich information about the selection history of populations, which cannot be obtained from classical selection scan approaches based on present time data only. Here we apply a new statistical approach taking advantage of genomic time series and a state of the art statistic (nSL) based on present time data to disentangle both old and recent signatures of selection in the Asturiana de los Valles cattle breed. This local Spanish originally multipurpose breed native to Asturias has been selected for beef production over the last few generations. With the use of SNP chip and whole-genome sequencing (WGS) data, we detect candidate regions under selection reflecting the effort of breeders to produce economically valuable beef individuals, e.g., by improving carcass and meat traits with genes such as *MSTN*, *FLRT2*, *CRABP2*, *ZNF215*, *RBPMS2*, *OAZ2*, or *ZNF609*, while maintaining the ability to thrive under a semi-intensive production system, with the selection of immune (*GIMAP7*, *GIMAP4*, *GIMAP8*, and *TICAM1*) or olfactory receptor (*OR2D2*, *OR2D3*, *OR10A4*, and *0R6A2*) genes. This kind of information will allow us to take advantage of the invaluable resources provided by gene bank collections from local less competitive breeds, enabling the livestock industry to exploit the different mechanisms fine-tuned by natural and human-driven selection on different populations to improve productivity.

## Introduction

Asturiana de los Valles is a Spanish cattle breed native to Asturias, in the north-western region of Spain.^1^ Being originally a multipurpose breed, it was selected for beef purposes over the last few generations. To this aim, the selection of homozygous individuals for a disruptive mutation in the myostatin (*MSTN*) gene, associated with the muscular hypertrophy phenotype (Dunner et al., 2003), has led to a remarkable increase in the frequency of the nt821(del11) mutation in Asturiana de los Valles, as shown by a 93.6% frequency found in the animals belonging to the last generation (those born between 2014 and 2020, Aseava unpublished data). Nowadays, the cattle are raised mainly under semi-intensive management conditions, ranging from evergreen pasturelands to harsh mountainous territories, and has broadened its geographical distribution to half of the Spanish territory, counting more than 60,000 individuals.

In recent decades, substantial efforts have been made for conserving animal genetic resources to ensure the adaptability of livestock production systems (Paiva et al., 2016). New technologies are creating novel opportunities in this field by increasing information on livestock genomes and tools that can be used to tackle global problems derived from population growth, income, and climate change (Bruford et al., 2015). Gene banks allow for *in vitro* conservation of substantial inventories of germplasm and tissues. They have emerged as invaluable resources for biodiversity and scientific research (Groeneveld et al., 2016), including reconstituting and enhancing the genetic variability of breeds (e.g., Blackburn et al., 2014; Kim et al., 2015). Far from representing breeds for one fixed point in time, gene bank collections have been shown to capture more diversity than some *in-situ* populations thanks to periodic resampling (e.g., Yue et al., 2015; Paiva et al., 2016).

Among gene bank applications, the genomic analysis of samples allows for inferences about recent natural and artificial selection signatures. Selection tends to cause specific changes in the patterns of genetic variation at both selected and neutral linked loci. Thus, using molecular data corresponding to present time individuals may identify signatures left by past events of positive selection in the genetic diversity of a population. In contrast to genome-wide association studies, the phenotypic response influenced by a candidate locus is unknown and must be deduced from the function of genes or transcripts found in the region and/or the selection constraints known to influence the population (which is often well documented in livestock populations; Larson and Burger, 2013). However, these constraints are also known to have varied along time, so hypotheses about the function selected at a given locus may strongly depend on the onset and intensity of its selection, which is difficult to estimate from present time data (Chen and Slatkin, 2013).

In this context, the analysis of samples from different time points available in gene banks promises to greatly improve the annotation of selection signatures, as this provides direct access to the temporal evolution of allele frequencies and might therefore indicate the time periods where an allele was selected (Malaspinas, 2016). In particular, gene bank data collected in the few last decades might allow us to distinguish alleles that have been selected as a result of recent selection objectives from those that had been selected before this period of modern intensive breeding. To illustrate this approach and detect selection using either temporal or present time sampling, we combined SNP chip data from previous projects and whole-genome sequencing (WGS) data for the Asturiana de los Valles breed produced within the European Project IMAGE, and built a dataset covering eight generations of this population. These data were used to detect selection signatures in this breed using both a new statistical approach taking advantage of genomic time series (Paris et al., 2019) and a state of the art statistic (nSL) based on a single sampling time (Ferrer-Admetlla et al., 2014). Apart from expanding our understanding on the genomic grounds of Asturiana de los Valles evolution and providing molecular tools for enhancing the performance of this breed, this study outlines one potential use of gene bank collections in animal breeding.

## Materials and Methods

### Samples

**Figure 1:**
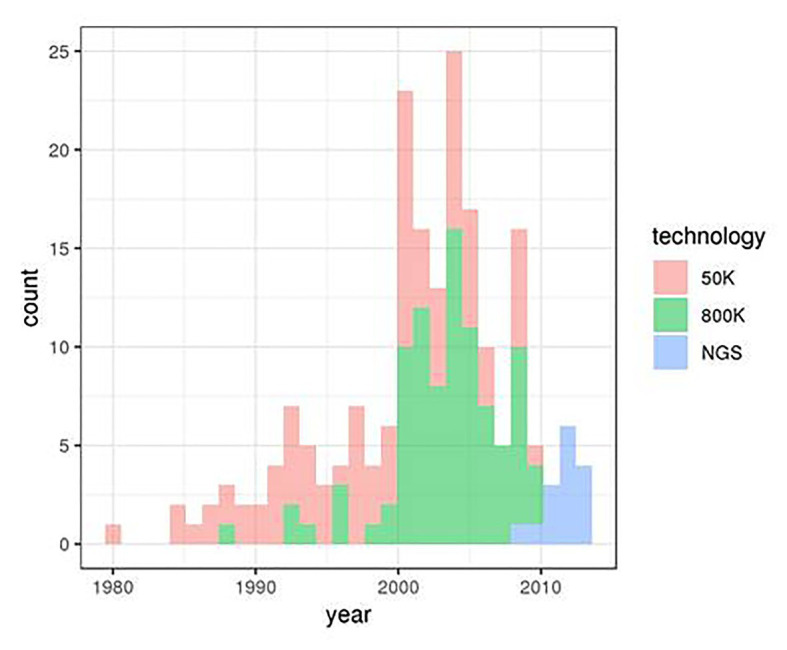
Distribution of Asturiana de los Valles birth dates in the three original data sets.

We considered genotype data from 153 animals of the Asturiana de los Valles bovine breed. These genotypes were obtained from three projects involving three distinct genotyping technologies: 88 sires were genotyped using the Illumina’s BovineSNP50 v. 2 chip within the Climgen project (FACCE_20171212), 50 animals (25 sires and 25 dams) were genotyped using the Illumina’s Bovine High Density BeadChip 770 k SNP within the Gene2Farm project (EU Seventh Framework Programme for research, technological development, and demonstration under grant agreement no. 289,592 – Gene2Farm), and WGS data were obtained for 15 sires within the IMAGE project (Innovative Management of Animal Genetic Resources. European Grant 677353). Animal birth dates ranged from 1980 to 2015, with different distributions for the three origins (Figure 1).

### DNA Sequencing and Bioinformatics Analysis

The 15 WGS samples were sequenced on a HiSeq 3,000, using 2 × 150 bp paired-end reads. The average coverage per animal ranged from 5.81 to 13.14, with a median value of 8.9. Sequences were mapped to the reference genome UMD3.1 using BWA v0.7.17 (Li, 2013). Optical and PCR duplicates were identified and marked using Picard tools v2.18.2 (Picard Toolkit, 2019). Local realignment around indels and base quality recalibration were performed with GATK (Van der Auwera et al., 2013). SNPs were then called using a two-step procedure. First, each sample was called independently using three alternative softwares: GATK HaplotypeCaller v3.7.0, samtools mpileup v1.8/bcftools v1.6 (Li, 2011), and FreeBayes v1.1.0 (Garrison and Marth, 2012). This provided two sets of variants: high-quality variants, which were found by the three callers and passed standard GATK quality filters, and low-quality variants that were found only in one caller and unfiltered. Second, GATK SNPs were filtered using the Variant Quality Score Recalibration (VQSR) of GATK, a machine learning algorithm that sets the quality filter thresholds based on two training datasets, respectively, representing true and spurious variants; these two training datasets were provided by the high-quality and low-quality variants obtained in step 1. A total of 15,768,037 autosomal bi-allelic SNPs and 302,181 bi-allelic SNPs on chromosome X were called from this procedure. In the next sections, we describe the different steps of the analysis for autosomal variants. Analysis of the X chromosome required specific treatments, which are described in the Supplementary Material.

### Merging and Cleaning Genotypes

In vcf files generated by GATK from WGS data, the quality of a genotype call for a given individual and variant is quantified by the value GQ = −10*log10(Pwrong), where Pwrong is the probability of this genotype call being wrong.^2^ For the WGS SNPs obtained as described in the previous section, individuals with genotype quality (GQ) below 10 (i.e., a probability of being wrong higher than 0.1) were set to missing, resulting in a relatively high rate of missing values per marker (about 23% on average). All variants with more than 40% missing values (1,439,050) were removed. Call rates were much higher in the SNP chip data sets; thus only SNPs with more than 5% missing values were removed, which provided 49,393 and 715,454 SNPs for the 50K and 770K datasets, respectively. The three datasets were finally merged using PLINK v1.9 (Chang et al., 2015), leading to a set of 35,656 autosomal SNPs with consistent positions and reference and alternative alleles in the three datasets. Genetic diversity at these markers is summarized by principal component analysis, which showed no significant effect of the genotyping technology (Supplementary Figure S1).

### Defining Temporal Samples

Generation time was set to 4 years based on the comparison between the birth dates of the 25 bulls of the Gene2Farm project used in our dataset (see Samples section) and the birth dates of 25 offspring of these bulls (one per bull) genotyped in the Gene2Farm project but not used in our study. Consequently, we divided the period 1980–2013 into nine consecutive non-overlapping periods of 4 years and defined these periods as the generations of the experiment. Animals were assigned to one generation according to their birth date. In order to satisfy the hypotheses of a Wright-Fisher evolution model, as assumed by the HMM time series approach, we then tried to limit inbreeding and relatedness within each generation by estimating the genetic relationship matrix in each generation using GCTA (Yang et al., 2011), focusing on SNPs with a minor allele frequency (MAF) greater than 10%. We removed animals with an inbreeding rate above 0.07 (six animals), and the most inbred animal of each animal pair with relatedness above 0.1 (30 animals). These two thresholds were based on a visual inspection of the empirical distributions of inbreeding and relatedness (Supplementary Figure S2) and aimed at removing outlier individuals or individual pairs. This led to a final set of 117 animals, with sampling times described in Supplementary Table S1. Only eight generations were used in the final analyses because generation 1 included no sample after filtering.

We also defined an alternative dataset without IMAGE’s WGS samples. Indeed, these animals represent a large part of the two most recent generations (8 and 9), so including them implies a focus on SNPs that are polymorphic over these two generations. However, SNPs that are monomorphic over generations 8 and 9 (removed by the WGS calling procedure described in DNA Sequencing and Bioinformatics Analysis section) might correspond to interesting selection signatures where one positively selected allele was fixed in the population before generation 8. Repeating the procedure described above without WGS samples led to a dataset including 43,951 SNPs and 106 animals belonging to generations 2–8 (Supplementary Table S1, last line).

In order to evaluate the potential impact of our choice of generation time, we also defined a dataset including all animals but classifying them into seven consecutive non-overlapping generations of 5 years. The resulting sampling times and sizes are shown in Supplementary Table S2.

### Detecting Selection From Time Series Data

We detected the loci that have been under selection in the Asturiana de los Valles breed between 1980 and 2013 using a new method that exploits the evolution of allele frequencies in a population along different sampling times (Paris et al., 2019). This method is based on a HMM approach, which allows for the modeling of both the stochastic evolution of population allele frequencies over time, as a result of genetic drift and selection (if any), and the additional noise arising from the finite sample size at each time point. Other similar HMM approaches were previously proposed in the literature, but they were either less accurate or limited for computational reasons to very small population sizes; see Paris et al. (2019) for more details. We applied the time series approach either with or without WGS samples (the number of individuals and SNPs for the two analyzed are summarized in Table 1); for SNPs that were shared by the two datasets, we kept the *p*-value computed with WGS samples, as this corresponds to the larger sample. A first look at the results showed four SNPs with extreme *p*-values, for which one allele was fixed in chip data while the other was almost fixed in NGS data. Such extreme patterns suggest an error (inversion of alleles) while merging the chip and NGS datasets, consistent with the fact that two of these SNPs were G/C SNPs. These were thus removed from the analysis. SNPs with a MAF below 5% over all the sampling times were also removed from this time series analysis. Indeed, some assumptions of the Likelihood Ratio Test used by Paris et al. (2019) to detect selection are not satisfied for rare alleles, which may lead to less accurate *p*-values. Besides, such SNPs correspond to allele frequency trajectories showing little variation over time, which are unlikely to contribute to significant evidence of selection in a time series analysis. This lead to a final set of 35,913 SNPs, among which 33,509 were segregating within WGS data and 2,404 were absent from these data.

**Table 1:** Summary of the datasets used for the selection scan.

Dataset	Nb. Indiv.	Nb. SNPs	Nb. HMM results	Nb. nSL results
All individuals	117	35,656	33,509	0
Without NGS individuals	106	43,951	35,913	0
Only NGS individuals	15	13,588,815	0	10,556,992

For each dataset, column “Nb. Indiv.” gives the number of individuals (after inbreeding and relatedness filters), column “Nb SNPs” gives the number of bi-allelic SNPs with required call rate, column “nb HMM results” gives the number of SNPs analyzed with the HMM approach (filtering out SNPs with MAF < 5% and likely merging errors), and column “nb nSL results” gives the number of SNPs with an nSL result. Note that all SNPs that were analyzed by the HMM based on all individuals (line 1) could also be analyzed by the HMM without NGS individuals (line 2); the HMM result considered for these SNPs in the manuscript was that based on all individuals.

In order to exploit linkage disequilibrium information, we detected genomic regions with a local excess of low *p*-values (i.e., of selection candidates), using the local score approach proposed in Fariello et al. (2017). The score function at each SNP was −log_10_(*p*-value)-1, as recommended by these authors to optimize detection power. As the distribution of *p*-values obtained from our test was close to uniform, we could evaluate the significance threshold for each chromosome using the closed-form formula provided in equation (3) of their study (p. 3703), implemented in the R code available at https://forge-dga.jouy.inra.fr/projects/local-score.

### Estimating Effective Population Size

Allele frequency trajectories not only depend on selection intensity but also on effective population size. Before estimating selection at each locus, we thus estimated this parameter by combining information from all loci. We used the method of Hui and Burt (2015) as implemented in the R package NB,^3^ and considered the dataset without the WGS samples to avoid bias against allele frequency trajectories where one of the alleles gets fixed before generation 8, which leads to an overestimation of population size.

### Detecting Selection From Present Time Data

We also applied a method that focuses on present time data and screens the genome for specific patterns kept by positive selection during population history, possibly a long time ago. Among the several methods available for this purpose, we computed the nSL statistic (Ferrer-Admetlla et al., 2014) using the software selscan (Szpiech and Hernandez, 2014). This statistic looks for long haplotypes segregating at high frequency in the population, measuring haplotype length by the number of SNPs rather than the genetic distance, which makes it more robust to local variations of the recombination rate.

To take advantage of the higher detection power derived from a higher SNP density, we computed nSL from WGS data using the following steps: (i) removing all variants with six missing genotypes or more; (ii) phasing the 15 individuals and imputing missing genotypes at the 13,588,815 remaining SNPs using shapeit (Delaneau et al., 2012); (iii) applying selscan to the phased and imputed haplotypes obtained at step (ii), which provided nSL scores at 10,556,992 SNPs (depending on local genetic diversity, the nSL score cannot always be computed); and finally, (iv) dividing SNPs into 20 bins according to their alternative allele frequency and standardizing nSL scores within each bin, using homemade R scripts. Following these different steps, candidate SNPs under positive selection are those lying in the tails of the distribution. In contrast to the time series test described above, the distribution of nSL under neutrality is unknown, so *p*-values cannot be easily computed. We, therefore, took an outlier approach, as proposed by Ferrer-Admetlla et al. (2014) in their analysis of human African populations. However, rather than defining the alternative allele as the derived one (based on outgroup information) and looking at candidate SNPs in both the lower and upper tail of the distribution, we defined the alternative allele in order to get a positive nSL score (during step iv) and looked at candidate SNPs only in the upper tail of the distribution.

## Results

### Selection Signatures Detected From the HMM Time Series Approach

The maximum likelihood estimation of effective population size in Asturiana de los Valles was equal to 408.3 animals, with a 95% confidence interval between 350 and 450. Based on this value, we evaluated the evidence for recent selection at 35,913 autosomal and 238 X-linked SNPs with a MAF above 5% over all the sampling times using the HMM time series approach. The smallest *p*-value was equal to 2.7e-05, which cannot be considered significant, given the number of tests performed. The *p*-value distribution was close to uniform (Supplementary Figure S3), as expected for any test under the null hypothesis, though with a deficit of very small *p*-values. This indicates that our testing procedure is well-calibrated while outlining that the dataset considered here presents little evidence for selection at the SNP level. However, when also accounting for the genomic position of tested SNPs using a local score approach, we could detect five candidate genomic regions under selection, i.e., with a significant excess of low *p*-values, for a chromosome-wide type I error rate of 10% (Table 2). Given that 28 chromosomes were analyzed, the expected number of false-positive signals for such a type I error rate is 2.8 genome-wide. Thus, we cannot exclude that some of the five regions detected are false positives, but we note that three of them were also detected for type I errors of 1 or 5% (Table 2).

**Table 2:** Candidate genomic regions under selection in Asturiana de los Valles since 1980, based on the HMM time series approach.

Chr	Start (bp)	End (bp)	Length (kbp)	Nb SNP	Signif	Genes
10	45,387,461	45,564,676	177	7	1%	*PLEKH02*, *PIF1*, ***RBPMS2***, ***OAZ2***, ***ZNF609***, ***RF00413***, and *TRIP4*
13	41,414,256	41,529,941	116	3	5%	-
17	4,675,045	4,750,693	76	4	10%	***FHDC1***, ***ARFIP1***
17	31,268,164	31,632,465	364	8	10%	-
22	39,414,833	39,491,373	77	3	5%	***PTPRG***

Single SNP *p*-values were cumulated using a local score approach, for a chromosome-wide type I error rate of 1, 5, or 10% (see the Signif column). Genes located less than 100 kb away from each region are indicated, and are in bold if included in the region.

In order to evaluate the influence of the generation time on these results, we repeated the time series analysis using a generation time of 5 years, focusing on SNPs found both in WGS and chip data. Single SNP *p*-values obtained using four or 5 years per generation were highly correlated (Supplementary Figure S4).

### Selection Signatures Detected From Present Time Data

Among the SNPs included in the HMM time series approach, 30,649 autosomal and 190 X-linked SNPs could be analyzed with the nSL procedure described in the Materials and Methods section. Because the *p*-values associated with a given nSL score is difficult to evaluate, we used an outlier approach considering all SNPs with an nSL score above 5 as potential candidates (see Supplementary Figure S5 for the full distribution of nSL scores). This approach provided eight candidate SNPs under selection, which could be grouped into six regions (Table 3).

**Table 3:** Candidate genomic regions under historical selection in Asturiana de los Valles detected by the nSL approach from SNPs genotyped in all generations.

Chr	Start (bp)	End (bp)	Length (bp)	Nb SNP	log_10_(pval)	Genes	Miss
2	7,169,804	7,270,116	100,312	2	9.82	***COL5A2***, *COL3A1*	5 & 5
2	8,476,975	9,202,511	726,536	2	8.12	***CALCRL***	0 & 5
6	55,360,713	-	-	1	7.85	-	2
7	20,631,252	-	-	1	6.90	*TICAM1*, *FEM1A*, *DPP9*, *MYDGF*, and *TNFAIP8L1*	5
10	98,290,813	-	-	1	10.10	*FLRT2*	2
25	13,647,777	-	-	1	7.10	*PARN*, *BFAR*, and *PLA2G10*	2

SNPs with an nSL score above 5 are shown and are grouped into one region if their physical distance is below 1 Mbp. Genes located less than 100 kb away from each region are indicated, and are in bold if included in the region. Column “miss” gives the number of missing genotypes per SNP in each region.

To take advantage of the higher SNP density available for this test (all recent samples were sequenced rather than just genotyped on a chip), we also considered the nSL results obtained for 10,556,992 autosomal and 168,022 X-linked SNPs called from WGS data. As expected, this higher SNP density provided a much higher detection power, retrieving 4,217 SNPs with an nSL score above 5, which could be grouped into 307 regions. Considering that isolated outstanding nSL scores are unlikely for such a high SNP density and might be due to false positives rather than true selection events, we focused on regions with more than 10 candidate SNPs and reduced this first list to 42 candidate regions, as reported in Supplementary Table S3. Six of them were particularly outstanding, as they exhibited more than 10 SNPs with an nSL score above 6. These six regions, listed in Table 4, include three of the top regions detected with nSL from the merged SNP chip-WGS dataset (Table 3), on chromosomes 2 (two neighboring regions that could be considered as one) and 10.

**Table 4:** Strongest candidate genomic regions under historical selection in Asturiana de los Valles, detected by the nSL approach from whole-genome sequencing (WGS) data.

Chr	Start (bp)	End (bp)	Length (kbp)	Nb SNP	Genes	Miss
2	6,550,846	9,649,084	3,098	43	*PMS1*, *ORMDL1*, *OSGEPL1*, ***ANKAR***, ***ASNSD1***, ***SLC40A1***, ***WDR75***, ***COL5A2,*** ***COL3A1***, ***GULP1***, ***CALCRL***, ***ZSWIM2***, ***FAM171B***, and *ITGAV*	2.57
3	13,815,189	14,308,800	494	11	*ETV3*, *ETV3L*, ***ARHGEF11***, ***LRRC71***, ***PEAR1***, ***NTRK1***, ***INSRR***, ***SH2D2A***, ***PRCC,*** ***HDGF***, ***MRPL24***, ***RRNAD1***, ***ISG20L2***, ***CRABP2***, ***NES***, ***BCAN***, ***HAPLN2***, ***GPATCH4***, ***NAXE***, ***TTC24***, ***IQGAP3***, and *MEF2D* (+1)	2.45
4	113,711,258	113,987,964	277	59	***GIMAP7***, ***GIMAP4*** and *GIMAP8* (+2)	2.63
10	94,233,010	94,292,043	59	19	-	2.74
10	96,843,755	98,709,965	1,866	161	***RF00019***, ***FLRT2***	2.65
15	46,464,438	46,728,630	264	511	*ZNF214*, *ZNF215*, *OR2D2*, *OR2D3*, ***OR10A4***, ***0R6A2***, and ***RF00026*** (+12)	2.19

Regions with more than 10 SNPs with an nSL score above 6 and a physical distance between consecutive SNPs below 1Mbp are shown (see Supplementary Table S3 for an extended list of candidate regions). Genes located less than 100 kb away from each region are indicated, and are in bold if included in the region. The number of genes without an ID is indicated in parenthesis. Column “miss” gives the average number of missing genotypes per SNP in each region.

Because these nSL results were based on partly imputed genotypes (see the Materials and Methods section), we also checked whether they could be biased by the proportion of imputed (i.e., initially missing) genotypes in a region. We found no evidence of such bias, as the proportion of imputed genotypes in SNPs from Table 3 and regions from Table 4 was not significantly higher (or lower) than the genome-wide average of 2.60 imputed genotypes per SNP.

### HMM Time Series vs. nSL Results

**Figure 2:**
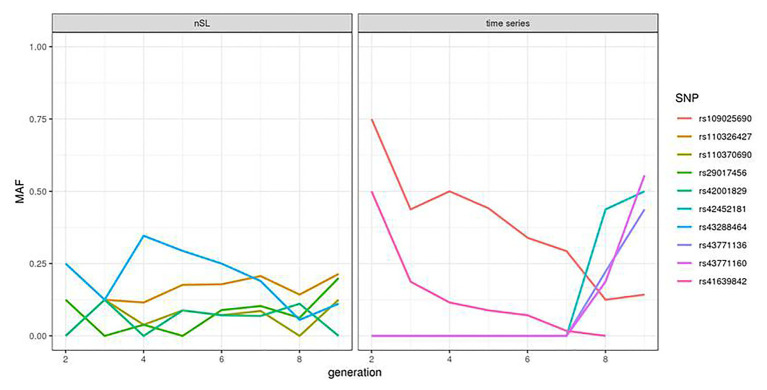
Allele frequency trajectory observed over the eight analyzed generations of the top five SNPs of the nSL and HMM time series tests.

We compared the statistics obtained from the HMM time series and nSL methodologies for each SNP where the two tests could be applied and observed very little correlation between the two signals (Supplementary Figure S6). For the five SNPs providing the highest nSL scores, the allele frequency trajectory in the last eight generations showed a constant and small value of the minor allele frequency (Figure 2, left). In contrast, at four of the five SNPs (rs109025690, rs109735272, rs41639842, and rs109105742) with the highest values of the HMM time series test, one allele segregating at an intermediate frequency (40–75%) in generation 2 became lost or very rare in generation 9; while for the last one (SNP rs41611975), one allele at frequency 0 in generation 2 increased up to 60% in generation 9 (Figure 2, right).

## Discussion

The present study, framed within the IMAGE project, aimed at enhancing the use of gene bank collections in animal breeding. To outline the invaluable resources provided by periodic resampling and cryopreservation of germplasm and tissue samples from local less competitive populations, we analyzed existing and new data from the Spanish Asturiana de los Valles beef cattle breed, using a new statistical approach that takes advantage of genomic time series to detect and characterize recent selection signatures in a population (Paris et al., 2019). Results from such selection scans will help the livestock industry to exploit the genetic variation fine-tuned by natural and human-driven selection on different breeds to improve productivity.

In the specific dataset considered here, only a few significant selection signatures were detected with this approach. Nevertheless, at least two of them included candidate genes potentially related to selection objectives in Asturiana (Table 2). Among the seven genes included in the Chr10 candidate region, *RBPMS2* is implicated in the bone morphogenetic protein pathway, *OAZ2* plays a role in cell growth and proliferation, and *ZNF609* is involved in myogenesis, all of them influencing the specific conformation of the double-muscled animals. The candidate region on Chr17 (4.7 Mb) included *ARFIP1*, a gene previously associated with milk yield and fat in Holstein (Lee et al., 2016).

Several non-exclusive statistical reasons may explain the limited number of detected regions. First, the experimental design (e.g., number of samples, number of generations) likely only allowed for the detection of SNPs under very strong recent selection. For instance, computer simulations performed in Paris et al. (2019, Figure 6) suggest that for an effective population size of 100 haploids and an evolution time of 10 generations, selected loci can be detected with reasonably high power only if selection intensity is greater than 0.5 (detection power should be higher for an effective size of 800 haploids as in Asturiana, but this scenario was not considered in the simulations). Second, only 35,913 SNPs were analyzed, which reduces the chance to observe markers in strong linkage disequilibrium with causal selected variants. The effect of SNP density on detection power has been outlined in previous selection scans for selection (e.g., Boitard et al., 2016). It can also be seen in the present study by the much lower number of candidate regions detected with the nSL score from the merged SNP chip-WGS dataset (30,649 autosomal SNPs) when compared with the WGS dataset alone (10,556,992 autosomal SNPs).

A more exhaustive deciphering of recent selection in the Asturiana breed, potentially revealing loci under weaker selection, could likely be achieved by including more samples and/or increasing SNP density using 800K chips or WGS data. Additional samples for the period considered in this study (1980–2013) are available in the Spanish cryobank. The time series could also be enriched by considering more recent samples, which may also improve detection power. However, no biological material is available before 1980, which corresponds to the creation of the bio-bank, so a retrospective time series analysis will not be possible before this date.

Another interesting observation from our study was the low correlation observed between the scores obtained from the time series and the nSL methodologies. While this might be due to the lack of power of the two tests in this specific dataset, this could also reflect a more fundamental complementarity between the two tests: the time series approach focuses on very recent selection events, and the nSL detects a larger variety of events, most of them being older than the period covered by our samples. Indeed, the allele frequency trajectory of the five SNPs providing the highest nSL scores in the last eight generations (Figure 2, left) suggests that the positively selected allele was already segregating at quite a high frequency at the starting point of our time series. The time series information contributes here to the annotation of selection signatures found by nSL: it reveals that in these five regions, most of the selection has likely been completed before 1980. In contrast, allele frequency trajectories associated with the five smallest *p*-values of the HMM time series test were characterized by a strong and almost monotonic variation (Figure 2, right). The absence of a strong nSL signal at these SNPs is more difficult to explain. In principle, strong nSL values are obtained when an initially very rare allele spreads in a population due to selection and reaches a frequency around 60–90% at the time where genomic data are collected. The five SNPs considered here could correspond to this situation, although for the four decreasing trajectories, this strongly depends on the shape of the trajectory further in the past. However, these SNPs are most likely not the causal variants under selection, and the allele frequency trajectory at the causal variants might be quite different from the ones observed here, especially with the low SNP density of our dataset.

One of the top regions detected by the nSL methodology, when considering either the SNPs shared by the chip and the WGS data or the WGS data alone, was located on Chr2 around 7 Mb (Tables 3 and 4). This region includes the myostatin (*MSTN*) gene, whose allele nt821(del11) associated with the double muscling phenotype, was probably introduced in the north of Spain in the 1940s through Simmental hypertrophic individuals, a trait inherited from Central European Frisian bovines (Garcia Fierro, 1972; Ménissier, 1982; Dunner et al., 2003). This characteristic was well accepted in the Asturiana de los Valles breed, where traditionally associated negative aspects such as dystocia, are kept below 2%, while displaying clear advantages which include increases in carcass yield (63% vs. 56% in wild-type), leaner muscle (85% vs. 77%), higher carcass conformation (14.1 vs. 9.1 - in a 1-15 score list rank), and fat scores (2.4 vs. 5.4).^4^ In the animals belonging to the last generation, the frequency of the mutated allele responsible for this trait is 93.6%, and mutated homozygotes are at an 89% frequency (Aseava unpublished information). In the MSTN gene neighborhood, the signature includes 14 other genes (see Table 4), probably swept by the effect of hitchhiking.

The nSL approach retrieved five other regions, including relevant functional candidate genes (Tables 3 and 4). A region on Chr4 included three GTPase genes (*GIMAP7*, *GIMAP4*, and *GIMAP8*), IMAP family members that have been related to the primary immunodeficiency pathway and were shown to play a major role in feed utilization and the metabolism of lipids, sugars, and proteins in Jersey cattle (Salleh et al., 2017). In line with these functions, a region on Chr 7 included the *TICAM1* gene, involved in native immunity and previously associated with bovine trypanotolerance in some African *Bos taurus* breeds (Noyes et al., 2011). Another region on Chr 10, detected with nSL on both the merged SNP chip-WGS dataset and WGS dataset alone, included the *FLRT2* gene, which is related to embryonic development (Haines et al., 2006) and has been associated with calf birth weight by a GWAS in Holstein (Cole et al., 2014). The gene *CRABP2* in the Chr 3 region has been also related to growth traits in beef cattle (Wen et al., 2020). Finally, the Chr15 region includes a cluster of olfactory receptor genes (*OR2D2*, *OR2D3*, *OR10A4*, and *0R6A2*), a family that is implicated in appetite regulation (Soria-Gomez et al., 2014) and for which genome-wide copy number variants have been associated with 10 diverse production traits in Holstein cattle (Zhou et al., 2018). This region also harbors *ZNF215*, an imprinted gene associated with growth and body conformation traits in Holstein cattle (Magee et al., 2010) and Beckwith-Wiedemann syndrome in humans, a genetic disorder characterized by growth abnormalities (Weksberg et al., 2010).

All these candidate regions may be driven by the recent selection of beef traits applied on Asturiana de los Valles since the middle of the past century. Muscular hypertrophy was selected through a handful of sires, and a founder effect cannot be ruled out, which may also explain other regions under selection in this breed. Also, the pleiotropic ability of the myostatin responsible for muscular hypertrophy has to be considered. This means that the presence of the mutation that disrupts the normal myostatin protein produces more effects than just the apparent excessive muscular growth and affects the activity of many key enzymes involved in fatty acid β-oxidation and glycolysis processes in cattle. Also, *MSTN* knockout triggers the activation of AMPK signaling pathways to regulate glucose and lipid metabolism by increasing the AMP/ATP ratio (Xin et al., 2019). The ability of *MSTN* to alter not only beef traits, but also meat and carcass quality, suggests a biological (rather than statistical) explanation for the particular scarcity of recent selection signatures in Asturiana de los Valles, where *MSTN* has fulfilled most of the plans of selection: few genomic regions were under strong selection in this breed because many phenotypic changes could be simultaneously obtained by acting on the *MSTN* gene alone.

However, it is plausible that some other regions may be under active selection and implicated in the process of breed differentiation and the development of the double-muscled phenotype, as highlighted in previous studies (Dunner et al., 2003; González-Rodríguez et al., 2017). This would allow us to interpret that the selection of traits such as feed conversion rate in Asturiana de los Valles, demonstrated by the increasing feed intake capability of the testing sires over the years, or the ability to produce good beef conformation, is advantageous. Also, selection of the olfactory receptor genes and immunity factors may be the result of maintaining the ability of this breed to thrive in a semi-intensive production system that includes 4 months outdoors in harsh mountainous territories above 2,000 mts, where cattle have to live in completely feral conditions under important predation pressure from wolf populations.

In conclusion, allele frequency trajectories over the few last generations contain rich information about the selection history of populations, which cannot be obtained from classical selection scan approaches based on present time data only. The HMM time series approach combined with a statistical method allowing for the detection of clusters of small *p*-values pointed out several candidate regions in the Asturiana de los Valles cattle breed with a clear shift in allele frequencies over the few last generations. It also allowed for annotating historical signatures found by the nSL statistic by showing that the advantageous allele in these regions was already at high frequency in the breed in 1980 and did not further expand over this time. The HMM time series and nSL signatures of selection included several candidate genes related to carcass and meat traits (*MSTN*, *FLRT2*, *CRABP2*, *ZNF215*, *RBPMS2*, *OAZ2*, and *ZNF609*), immunity (*GIMAP7*, *GIMAP4*, *GIMAP8*, and *TICAM1*), or olfactory receptors (*OR2D2*, *OR2D3*, *OR10A4*, and *0R6A2*), which inform us about the direction of applied active selection in the last few decades in Asturiana de los Valles. These results reflect the effort of breeders to produce economically valuable beef individuals while maintaining the ability to thrive under a semi-intensive production system. Overall, the outcomes from this study outline the critical resource for the understanding of breed history and the detection of relevant functional genes and variants provided by gene banks.

## Data Availability Statement

The SNP chip and WGS datasets newly generated for this study can be found in the European Nucleotide Archive (ENA, accession number PRJEB38981) repository. The SNP calling pipelines (DNA Sequencing and Bioinformatics Analysis) can be found at https://forgemia.inra.fr/bios4biol/workflows/-/tree/master/Snakemake. Pipeline IMAGE_calling was used for initial calling and pipeline IMAGE_vqsr for refined calling using the VQSR approach. The scripts implementing the other analyses described in the Materials and Methods section (Merging and Cleaning Genotypes, Defining Temporal Samples, Detecting Selection From Time Series Data, Estimating Effective Population Size, and Detecting Selection From Present Time Data) can be downloaded at https://github.com/sboitard/Asturiana_analysis.

## Ethics Statement

Ethical review and approval was not required for the animal study because it was based on available genomic data.

## Author Contributions

Project conception was performed by SB and SD. CP was a PhD fellow who worked on the development of the HMM software with the supervision of SB and BS, and SB also conducted the statistical analysis of the Asturiana data. NS and SD contributed to the genetic analysis of the candidate regions. KB-K performed the SNP calling analysis. The manuscript was drafted by SB, SD, and NS. All authors contributed to the article and approved the submitted version.

### Conflict of Interest

The authors declare that the research was conducted in the absence of any commercial or financial relationships that could be construed as a potential conflict of interest.

## Footnotes

^1^www.aseava.com

^2^https://samtools.github.io/hts-specs/VCFv4.3.pdf

^3^https://cran.r-project.org/web/packages/NB/

^4^https://www.aseava.com/raza_capitulo_10.aspx
