# Supplementary material for: Gene Banks as Reservoirs to Detect Recent Selection: The Example of the Asturiana de los Valles Bovine Breed
Source: Front Genet. 2021 Feb 2;12:575405. doi: 10.3389/fgene.2021.575405 (PMC7901938; doi:10.3389/fgene.2021.575405)
Supplement: Supplementary file 1 [file Table_1.docx]

Supplementary Material

**Supplementary Table S1.** Classification of Asturiana de los Valles samples into 9 generations, assuming a generation time of 4 years.

| **Generation** | **1** | **2** | **3** | **4** | **5** | **6** | **7** | **8** | **9** |
| --- | --- | --- | --- | --- | --- | --- | --- | --- | --- |
| First year | 1980 | 1984 | 1988 | 1992 | 1996 | 2000 | 2004 | 2008 | 2012 |
| Last year | 1983 | 1987 | 1991 | 1995 | 1999 | 2003 | 2007 | 2011 | 2015 |
| Initial sample size | 1 | 5 | 11 | 15 | 21 | 40 | 38 | 12 | 10 |
| Final sample size | 0 | 4 | 8 | 13 | 17 | 28 | 29 | 9 | 9 |
| Without WGS data | 0 | 4 | 8 | 13 | 17 | 28 | 29 | 7 | 0 |

**Supplementary Table S2.** Classification of Asturiana de los Valles samples into 7 generations, assuming a generation time of 5 years.

| **Generation** | **1** | **2** | **3** | **4** | **5** | **6** | **7** |
| --- | --- | --- | --- | --- | --- | --- | --- |
| First year | 1980 | 1985 | 1990 | 1995 | 2000 | 2005 | 2010 |
| Last year | 1984 | 1989 | 1994 | 1999 | 2004 | 2009 | 2014 |
| Initial sample size | 3 | 8 | 18 | 24 | 57 | 28 | 15 |
| Final sample size | 3 | 6 | 15 | 19 | 38 | 21 | 12 |

**Supplementary Table S3.** Candidate genomic regions under historical selection in Asturiana de los Valles, detected by the nSL approach from WGS data. Regions with more than ten SNPs with a nSL score above 5 and a physical distance between consecutive below 1Mbp are shown.

| **Chr** | **Start (bp)** | **End (bp)** | **Length (kbp)** | **Nb SNP** |
| --- | --- | --- | --- | --- |
| 1 | 17284145 | 17844420 | 560275 | 16 |
| 1 | 58941736 | 59087436 | 145700 | 12 |
| 1 | 93955160 | 94191385 | 236225 | 15 |
| 2 | 6183997 | 11164968 | 4980971 | 520 |
| 2 | 12202062 | 14569252 | 2367190 | 22 |
| 2 | 122118134 | 123089166 | 971032 | 10 |
| 3 | 9816274 | 11211962 | 1395688 | 58 |
| 3 | 12367374 | 14376749 | 2009375 | 84 |
| 3 | 62243313 | 62414854 | 171541 | 10 |
| 4 | 38254438 | 38370268 | 115830 | 14 |
| 4 | 103509824 | 104498265 | 988441 | 13 |
| 4 | 110325705 | 111503965 | 1178260 | 12 |
| 4 | 113655062 | 114065274 | 410212 | 500 |
| 4 | 115607420 | 116402197 | 794777 | 10 |
| 5 | 78254291 | 78645500 | 391209 | 22 |
| 6 | 38502644 | 38583783 | 81139 | 20 |
| 6 | 54469533 | 55613003 | 1143470 | 43 |
| 6 | 90241628 | 90408124 | 166496 | 59 |
| 7 | 22887378 | 24494913 | 1607535 | 12 |
| 7 | 107628871 | 108023308 | 394437 | 16 |
| 8 | 10212916 | 10568713 | 355797 | 11 |
| 8 | 101717616 | 104007856 | 2290240 | 22 |
| 8 | 107861381 | 108084403 | 223022 | 43 |
| 9 | 16623165 | 17999596 | 1376431 | 24 |
| 9 | 24916570 | 27412764 | 2496194 | 27 |
| 10 | 27708288 | 27763770 | 55482 | 15 |
| 10 | 93091979 | 94383559 | 1291580 | 99 |
| 10 | 96843755 | 99719963 | 2876208 | 121 |
| 12 | 75610646 | 76866668 | 1256022 | 14 |
| 14 | 24458524 | 24892252 | 433728 | 16 |
| 15 | 46448534 | 46772911 | 324377 | 1396 |
| 16 | 73067193 | 73221671 | 154478 | 18 |
| 17 | 23400557 | 23571882 | 171325 | 13 |
| 17 | 71724875 | 72495847 | 770972 | 25 |
| 19 | 24541400 | 24557402 | 16002 | 33 |
| 20 | 27569528 | 27699533 | 130005 | 28 |
| 22 | 5639814 | 7265109 | 1625295 | 24 |
| 22 | 8566678 | 9658215 | 1091537 | 11 |
| 23 | 34464900 | 36230719 | 1765819 | 28 |
| 28 | 5841070 | 5880362 | 39292 | 15 |
| 29 | 14161908 | 14857997 | 696089 | 13 |
| 29 | 35014046 | 35691511 | 677465 | 44 |

**Supplementary Figure S1.** Principal component analysis projection of animals genotyped using different technologies.


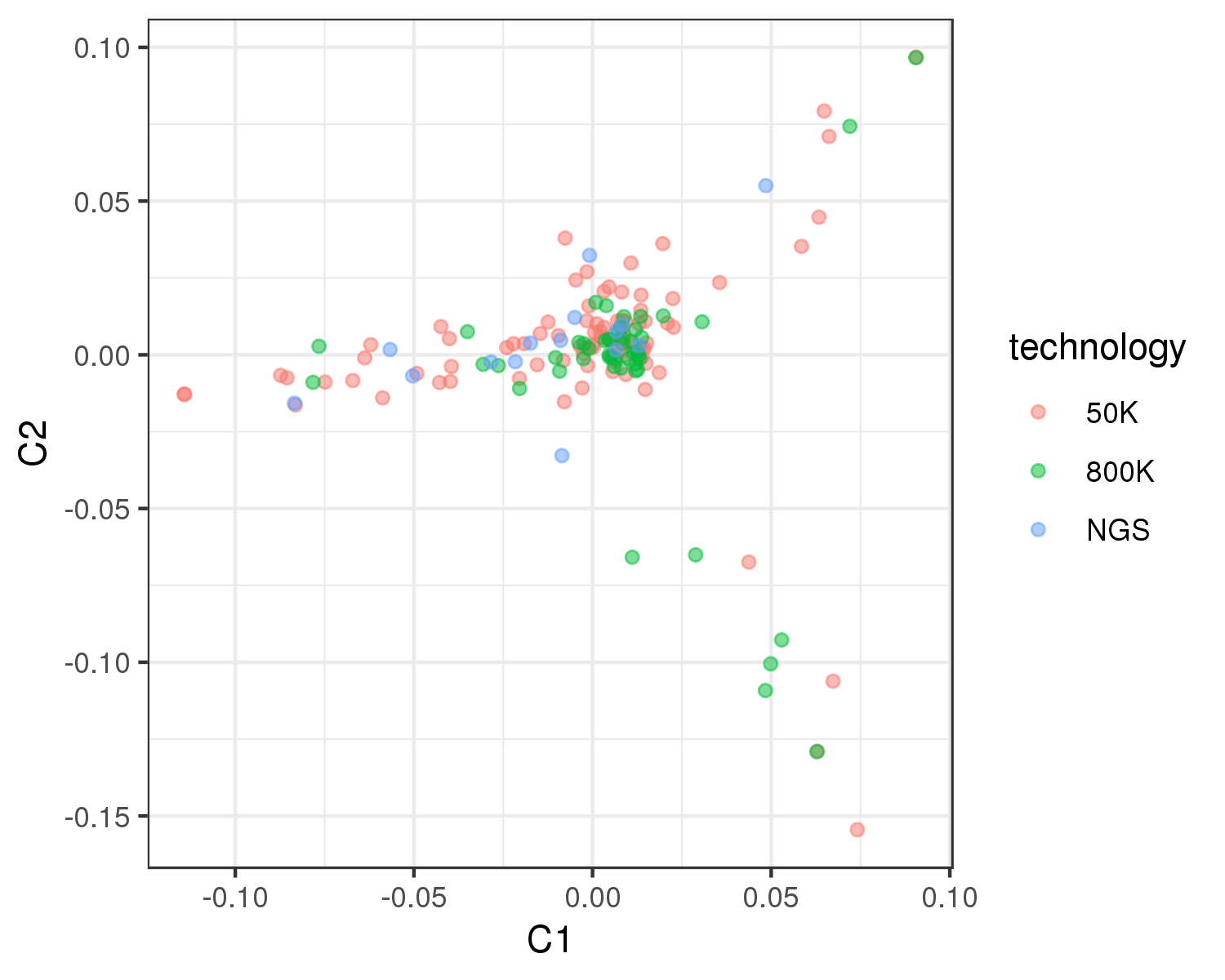


**Supplementary Figure S2.** Empirical distribution of genomic relationship within (left) and between (right) individuals among the 153 animals considered in this study, estimated from GCTA (Yang et al., 2011).


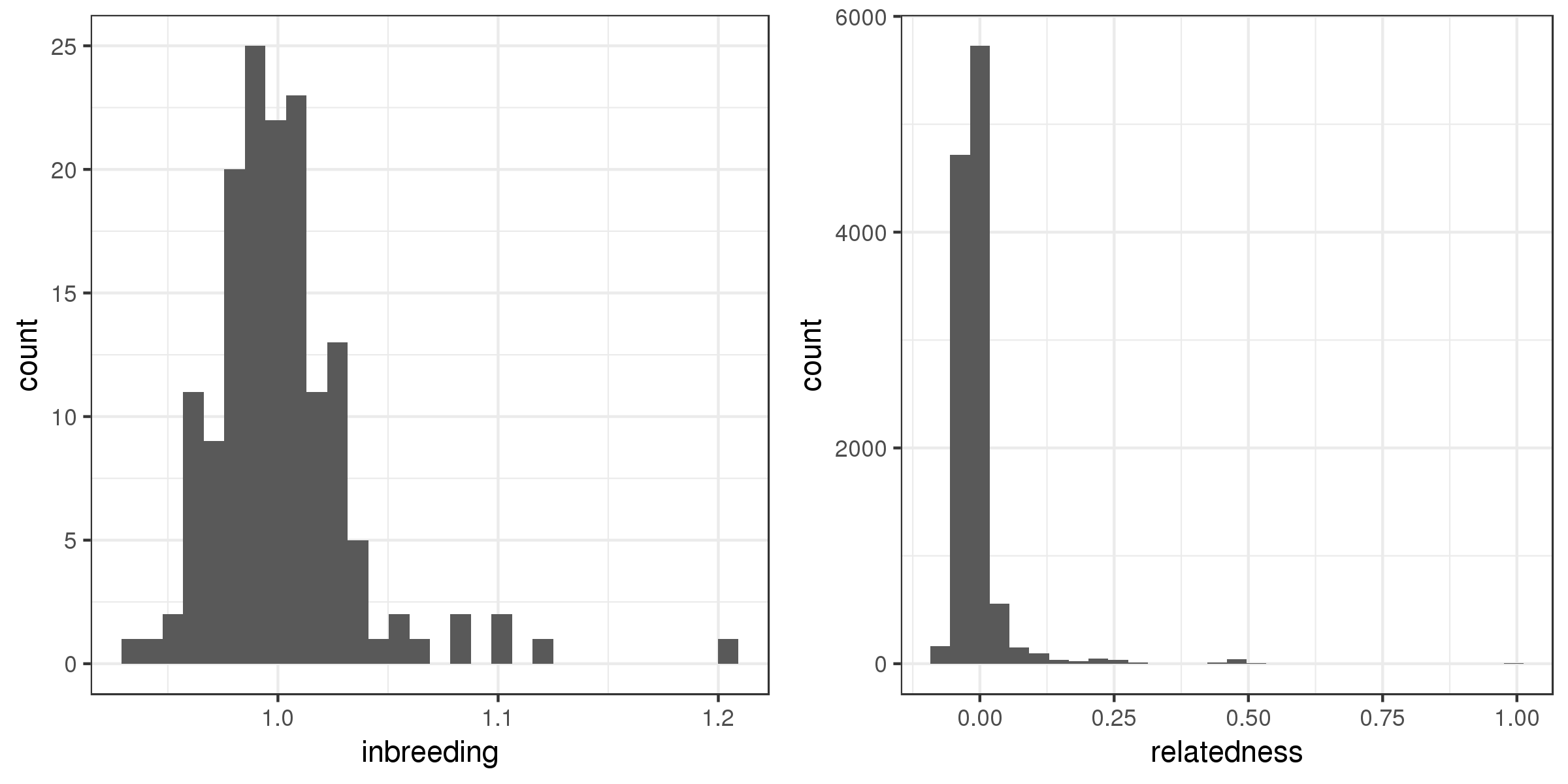


**Supplementary Figure S3.** Empirical distribution of HMM time series p-values in Asturiana de los Valles. For a given SNP, the p-value evaluates the probability of the observed allele frequency trajectory under neutral evolution.


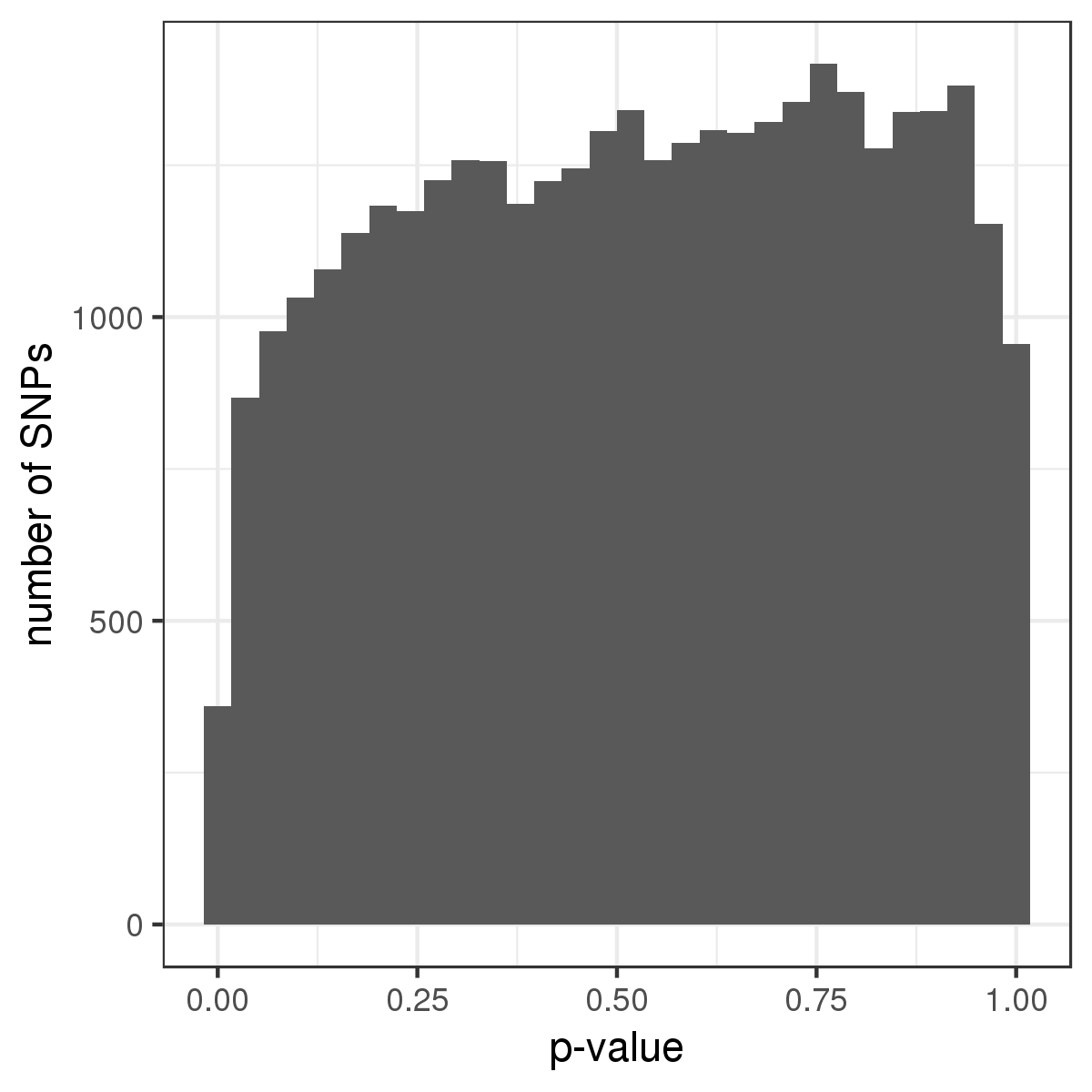


**Supplementary Figure S4.** Comparison of HMM time series p-values obtained at each SNP assuming a generation time of 4 or 5 years.


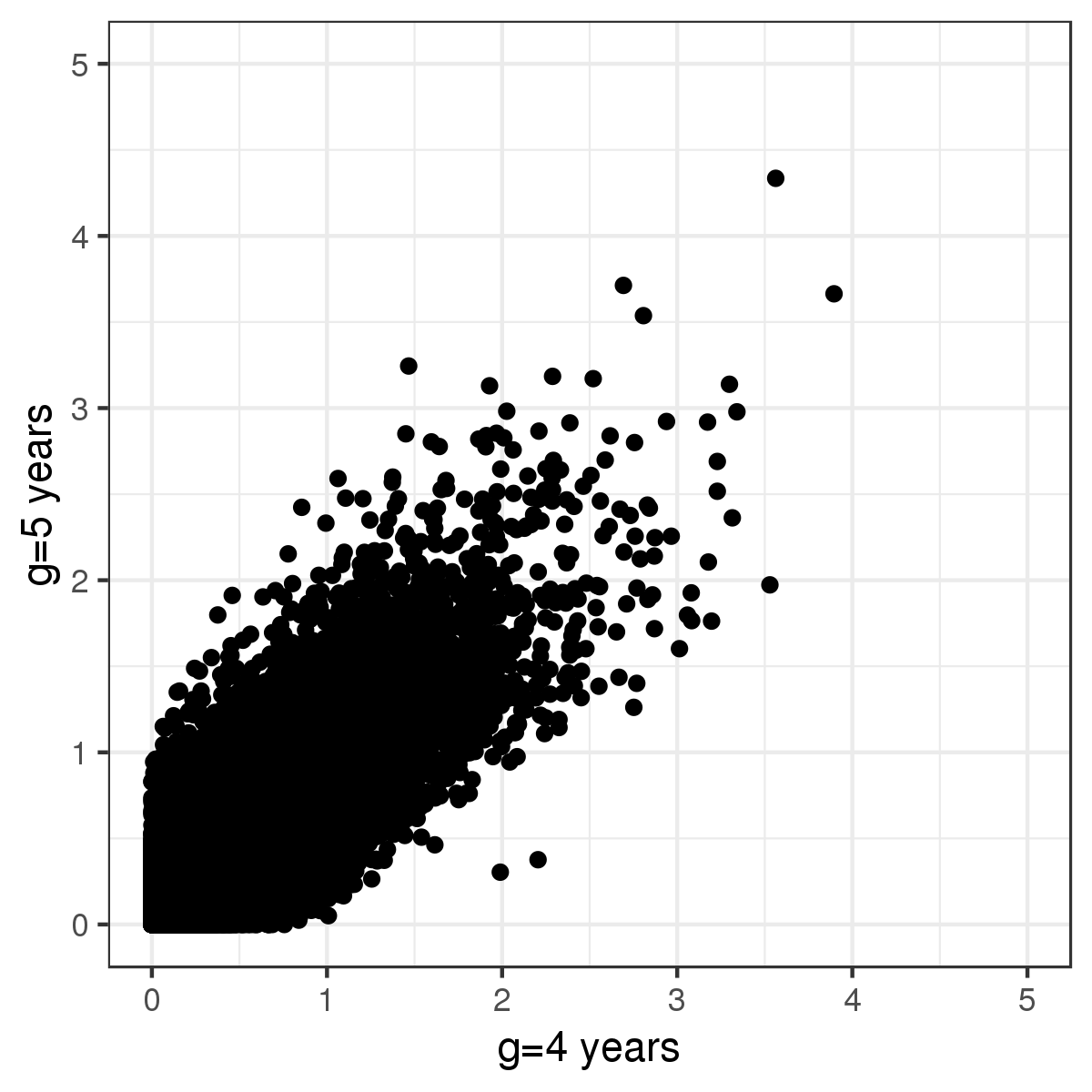


**Supplementary Figure S5** Empirical distribution of standardized nSL scores in Asturiana de los Valles for the merged SNP chip – WGS dataset.


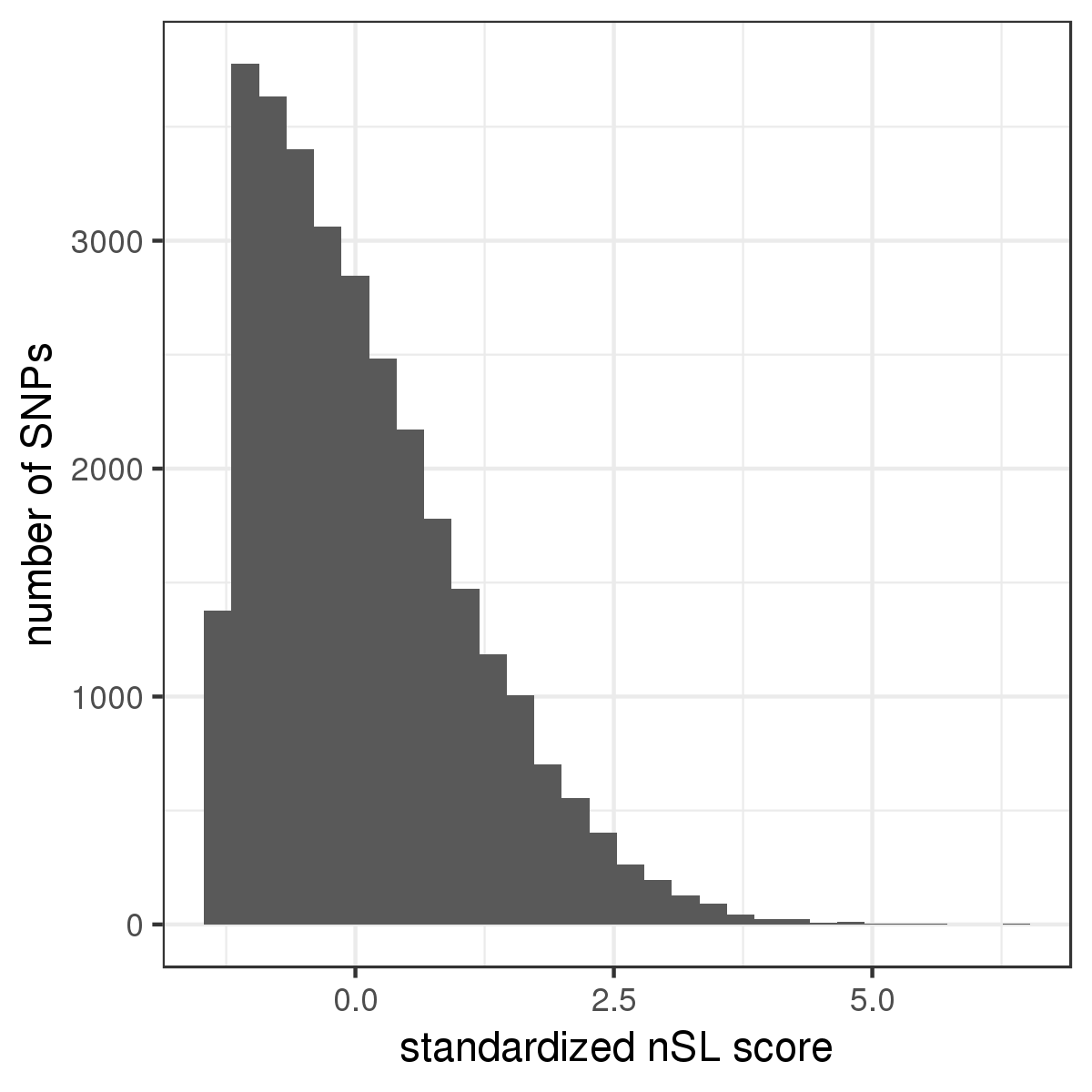


**Supplementary Figure S6.** Comparison of the selection statistics obtained from the HMM time series and nSL methodologies in Asturiana de los Valles for each SNP where the two tests can be applied.


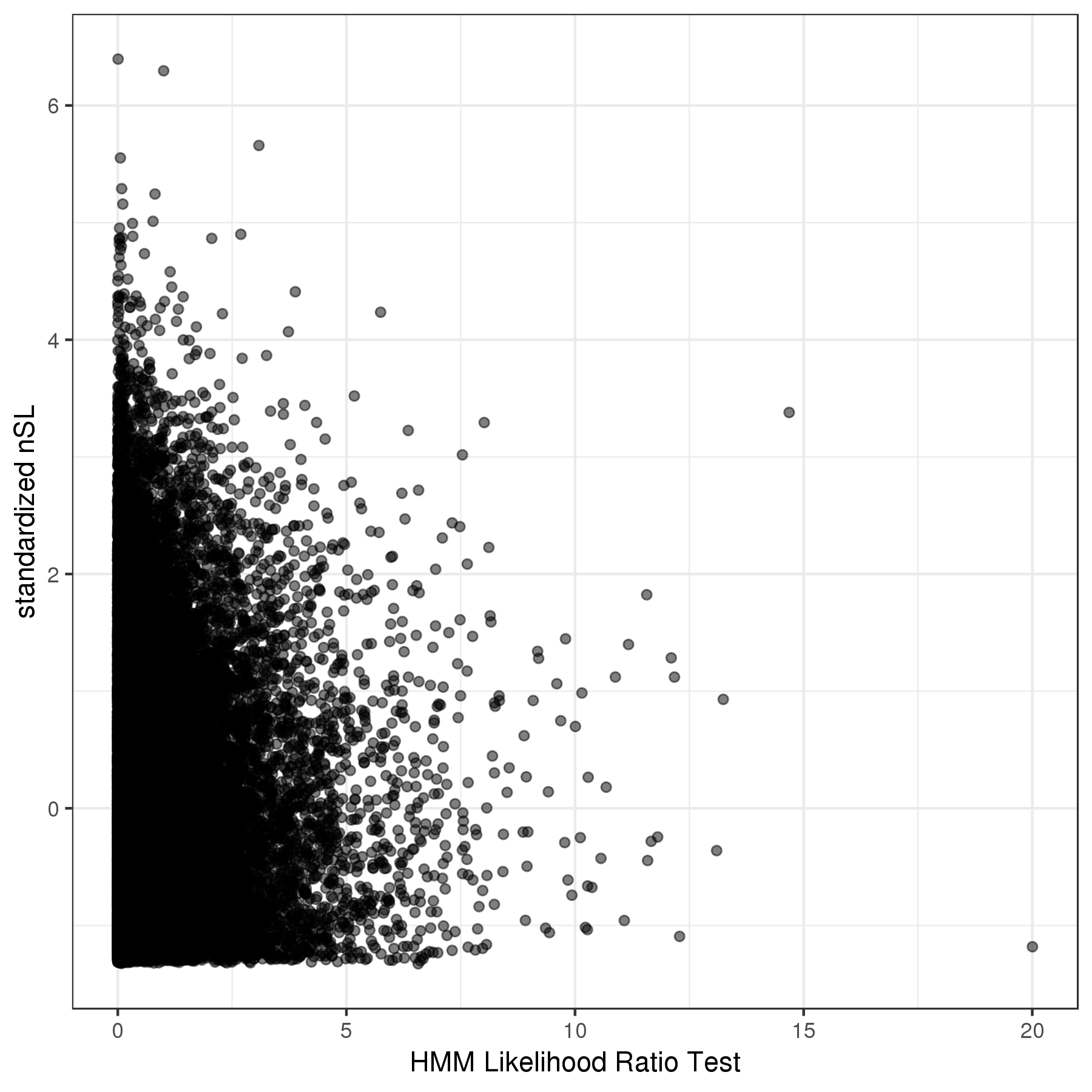


**Supplementary text: Analyses of the X chromosome**

Analysis of chromosome X focused on bi-allelic SNPs located from 0 to 137 Mb, excluding the pseudo autosomal region. When analyzing WGS data, heterozygous calls were set to missing because all 15 animals sequenced were males. Besides, due to the lower coverage expected for males on this chromosome, the genotype quality threshold was set to 5 (instead of 10 for autosomes). Combining these two filters (heterozygotes and genotype quality), 35% of genotype calls were missing on average. Removing markers with more than 40% missing values (as for autosomes) resulted in a set of 218,682 SNPs. SNP chip data were filtered using the same quality threshold as autosomal SNPs (less than 5% missing values), after also setting heterozygous calls to missing for all males. This provided 852 SNPs for the 50K dataset and 27,507 SNPs for the 800K dataset. Merging these three datasets and applying the same clustering / filtering of individuals as for autosomes lead to a set of 238 SNPs with a MAF above 5% that were analyzed with the HMM time series approach, among which 209 were segregating within WGS data. As this number is too low to obtain a reliable estimation of effective population size, we use the figure derived from autosomes for the time series analysis, applying a correction factor of ¾ corresponding to the proportion of X vs. autosomal chromosomes in a population under the assumption of a balanced sex ratio (Schaffner et al 2004).

The nSL analysis of the X chromosome was similar to that described for autosomes, with the 3 following excepions: (1) Genotype calls were set to missing using the procedure described in previous paragraphs. Applying the same call rate threshold as for the nSL analysis of autosomal markers, this lead to a set of 201,212 SNPs. (2) Option –chrX of shapeit was used. (3) Because two exactly identical haplotypes were returned by shapeit for each individual, only one of them was provided to nSL. A total of 168,022 nSL values were obtained by this procedure, among which 190 could be analyzed with the HMM time series approach.

**References:**

Schaffner, S. F. (2004). The X chromosome in population genetics. *Nature Reviews Genetics*, *5*(1), 43-51.
